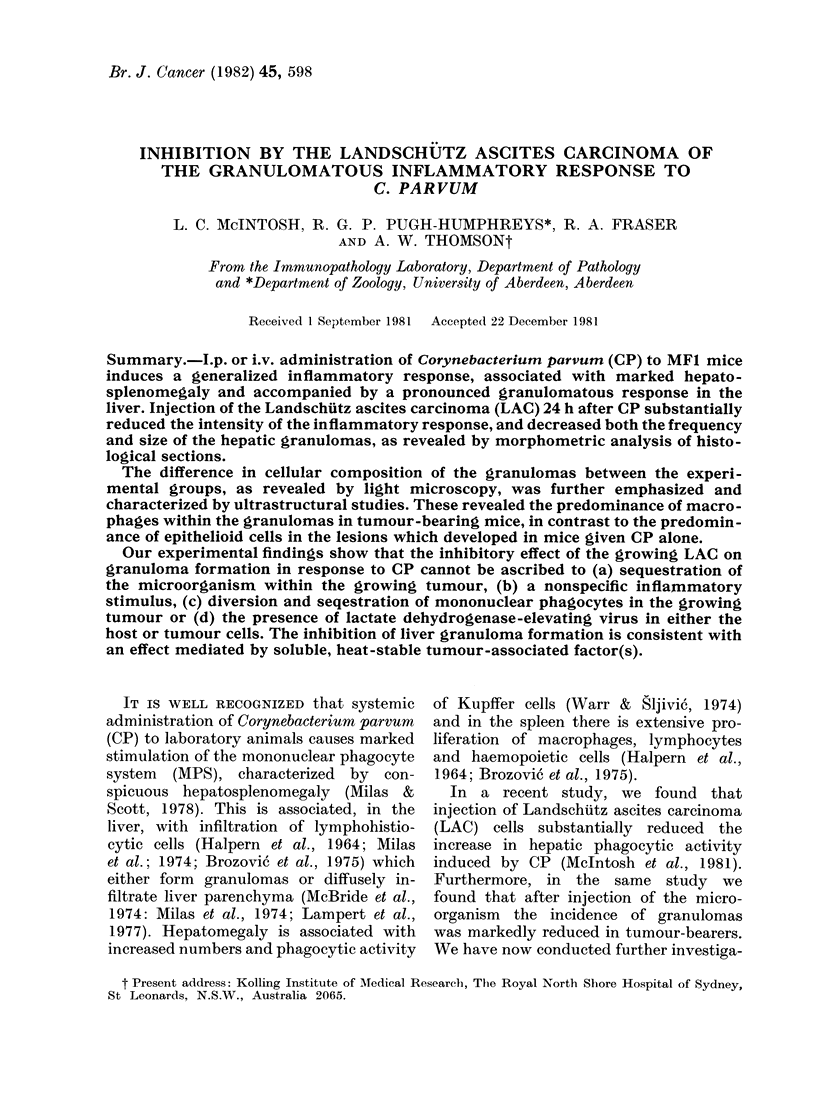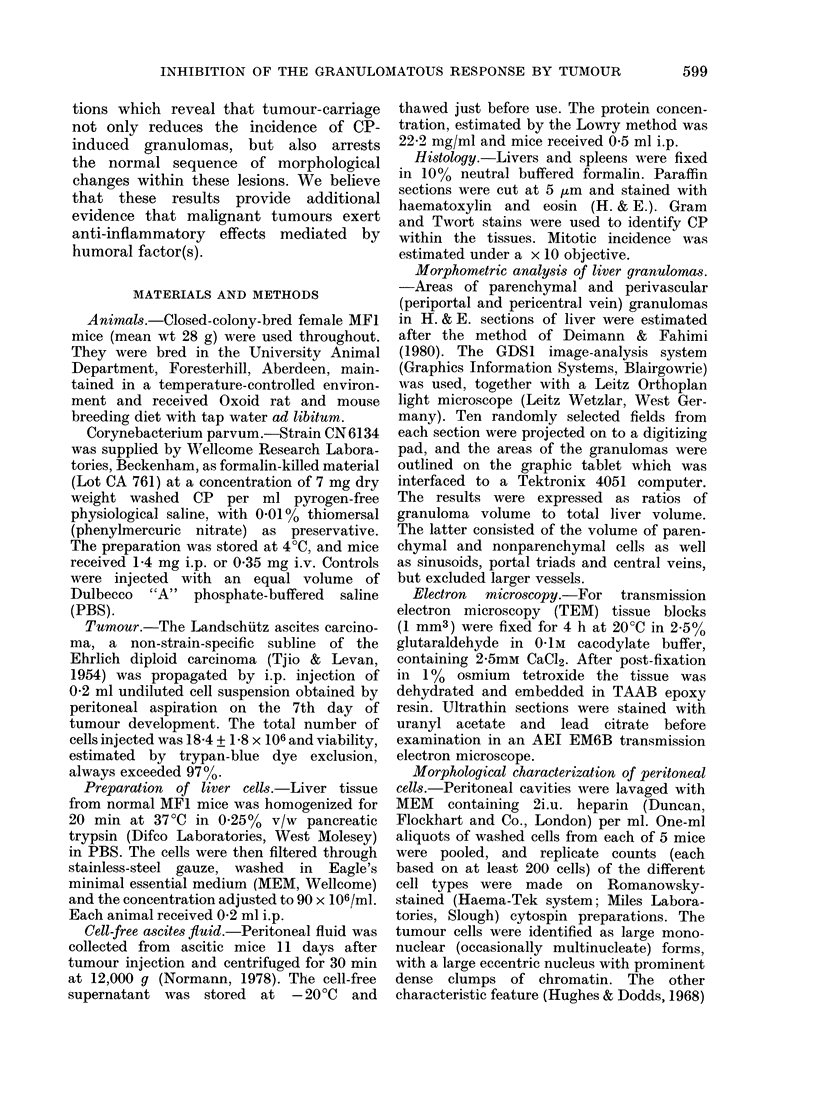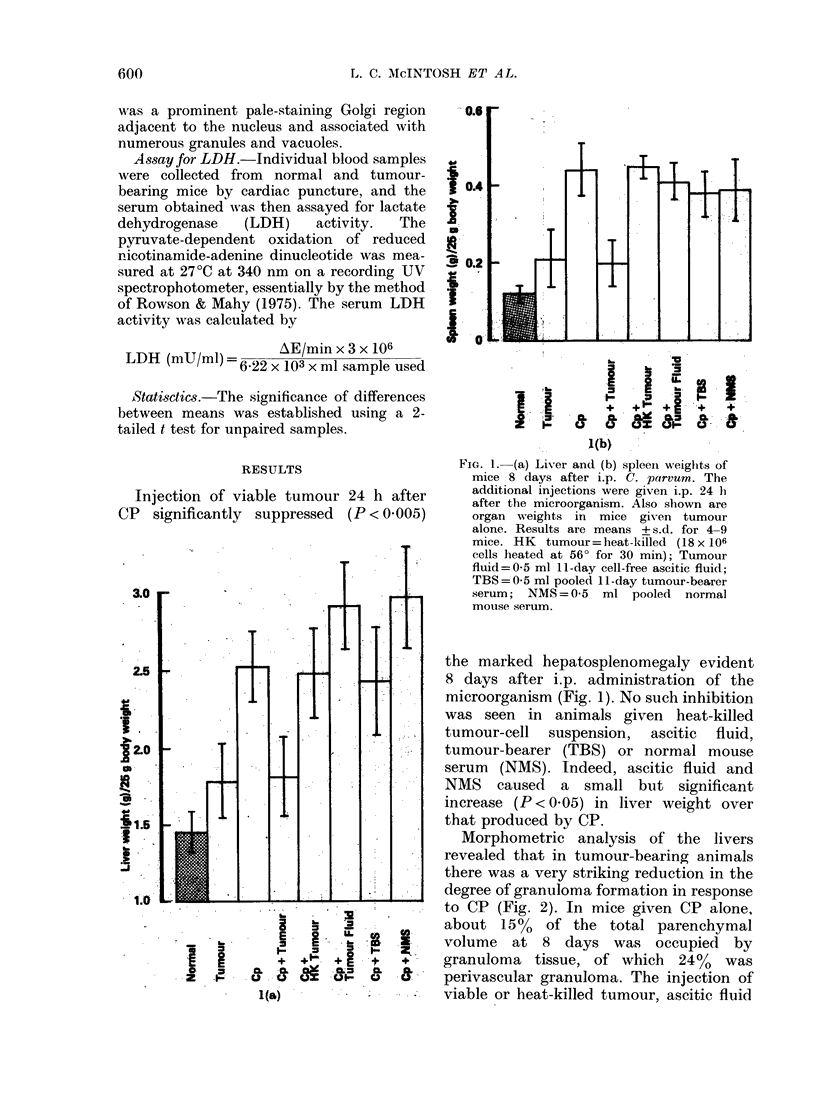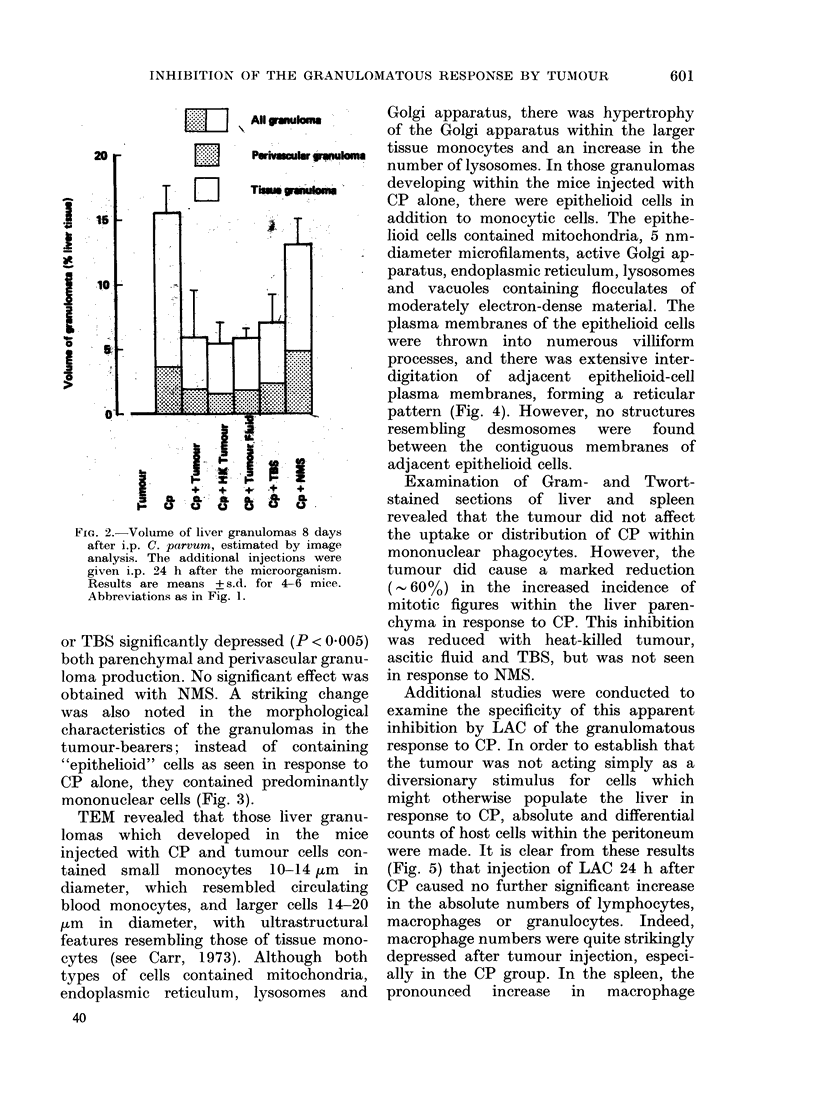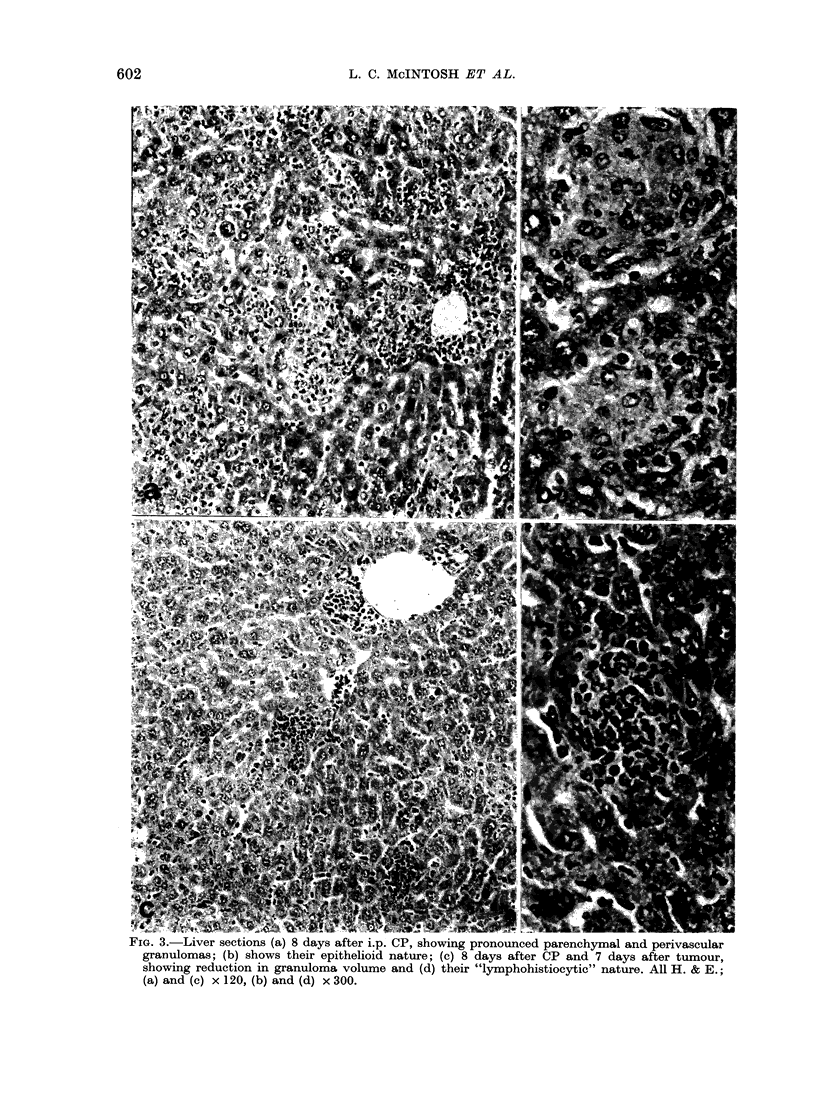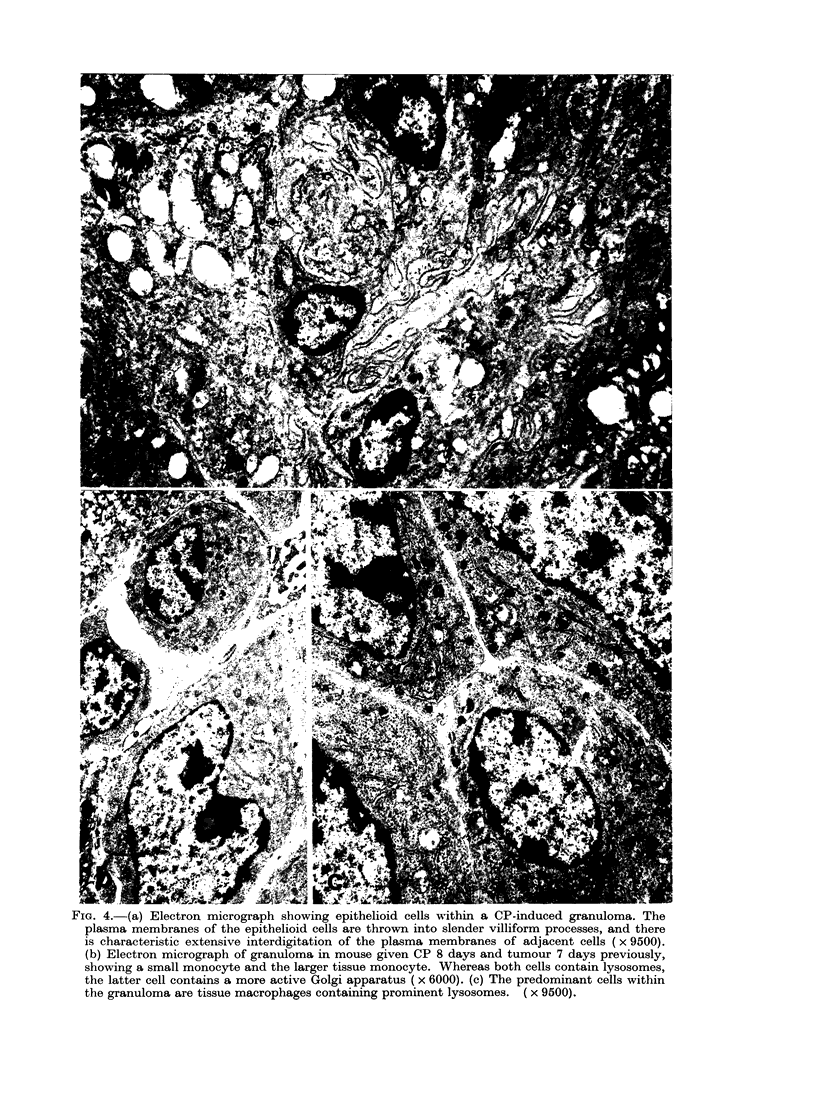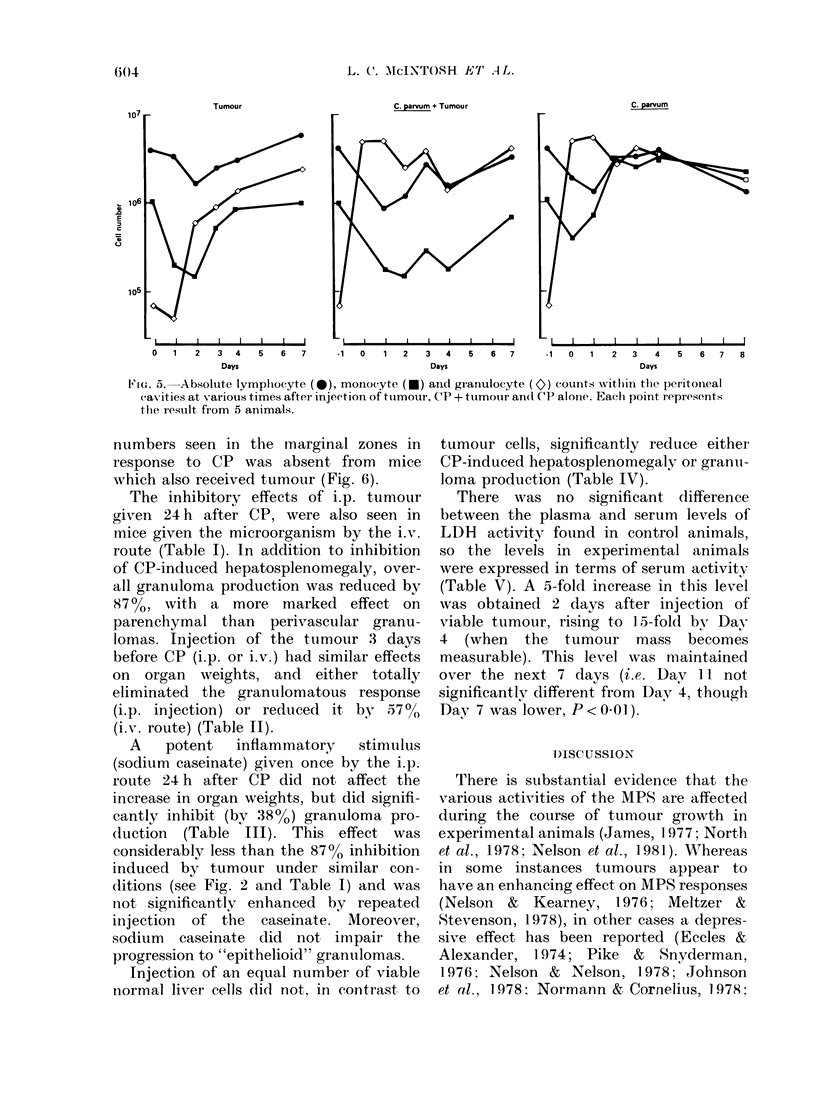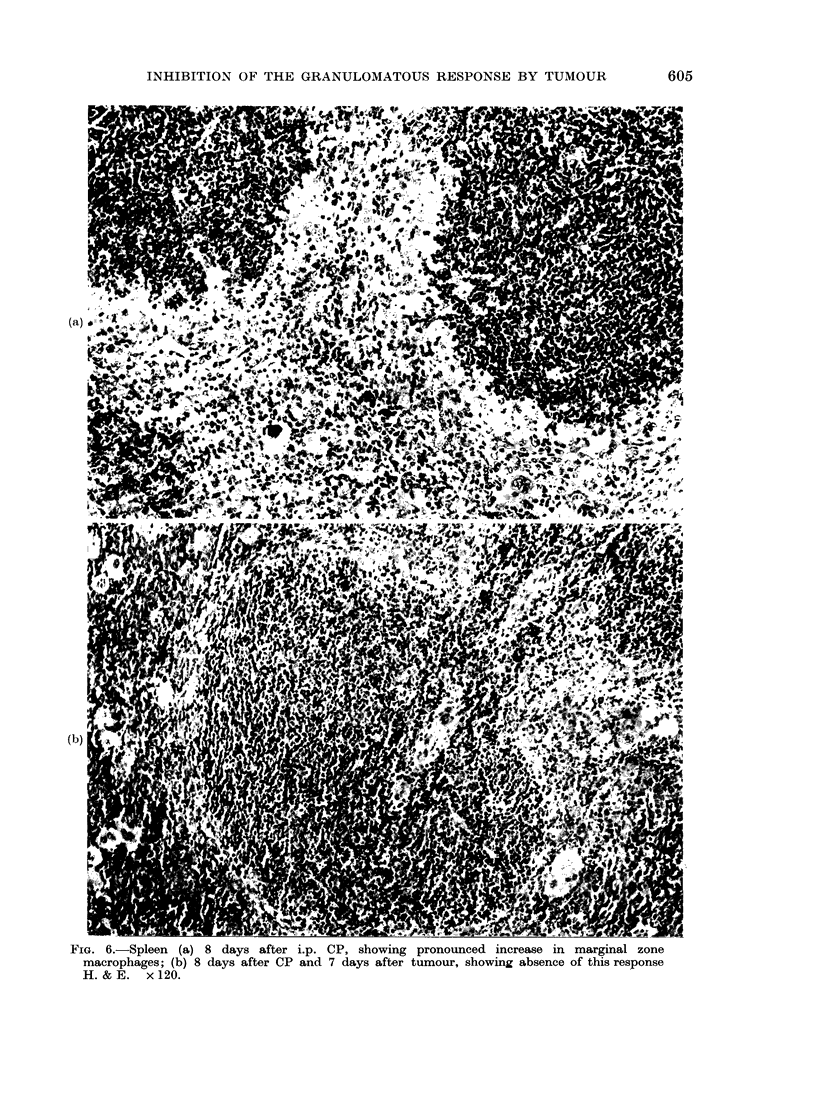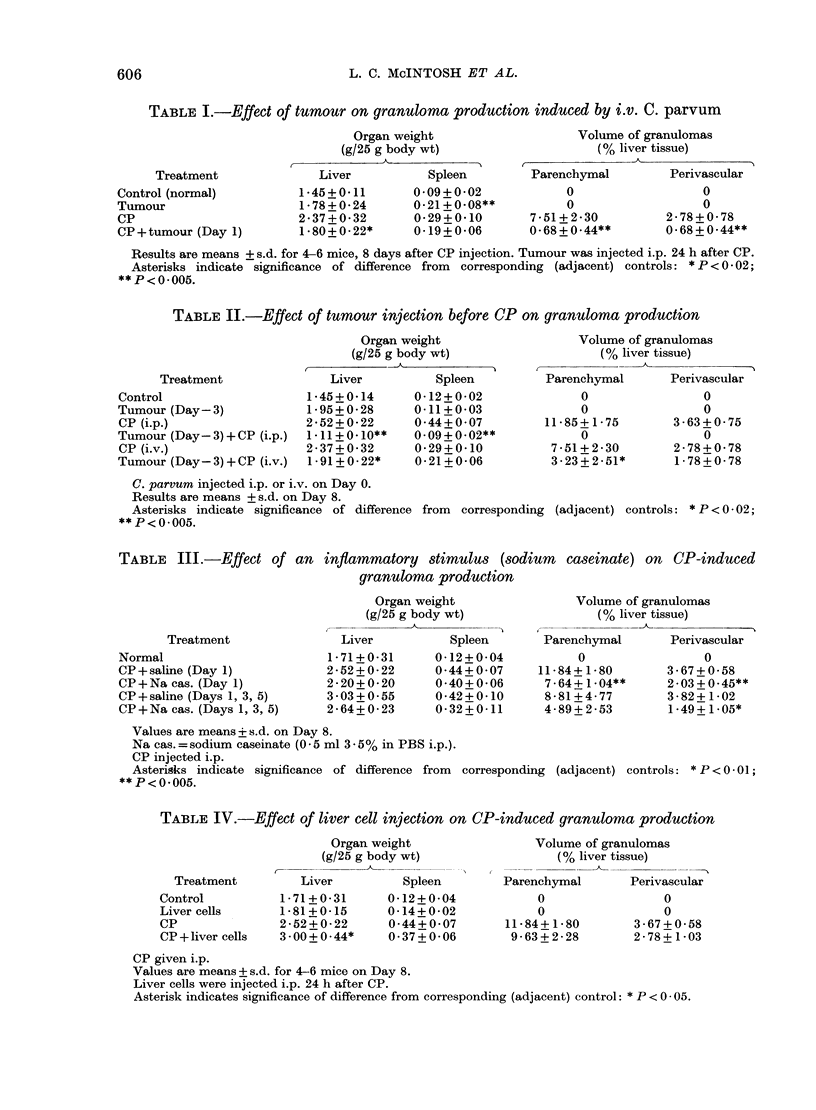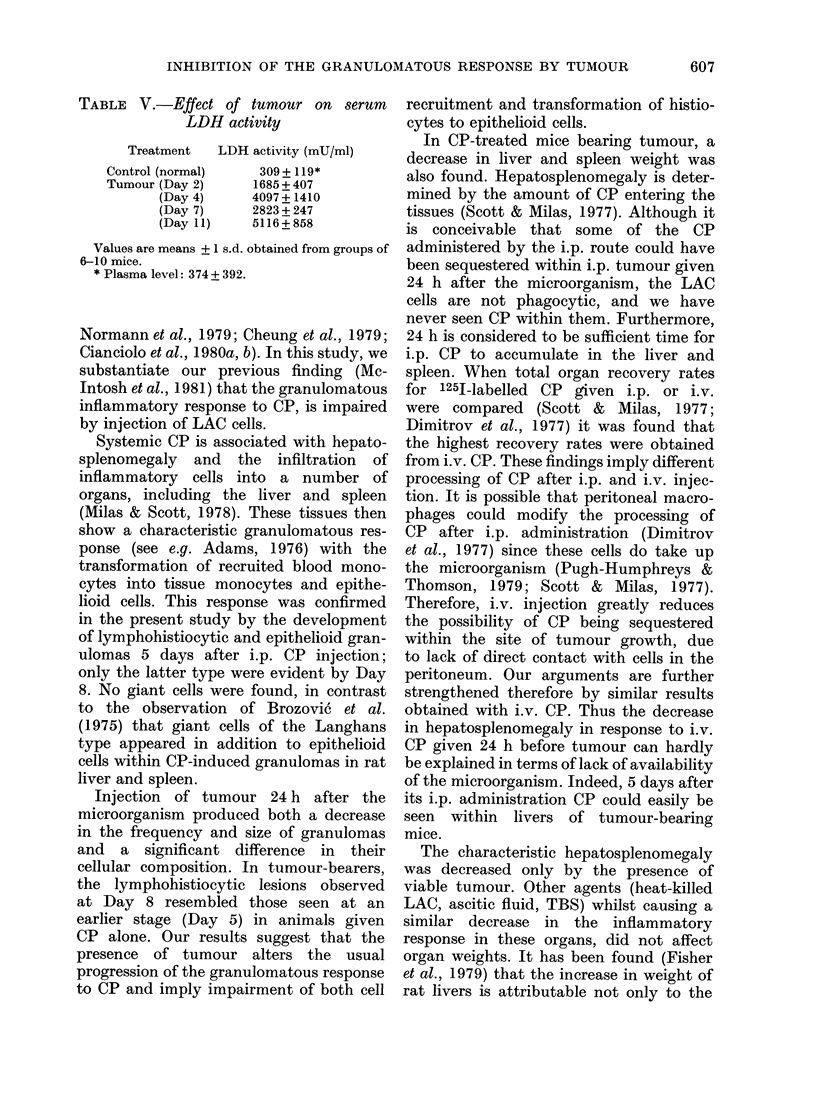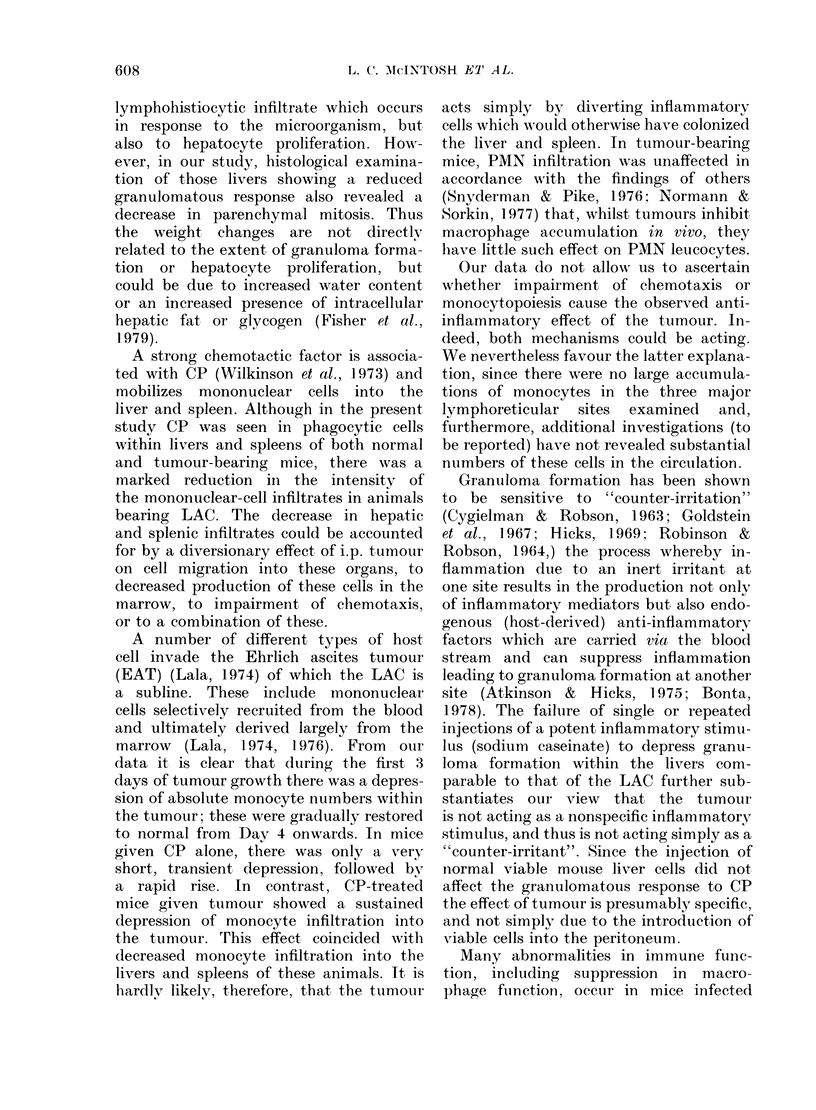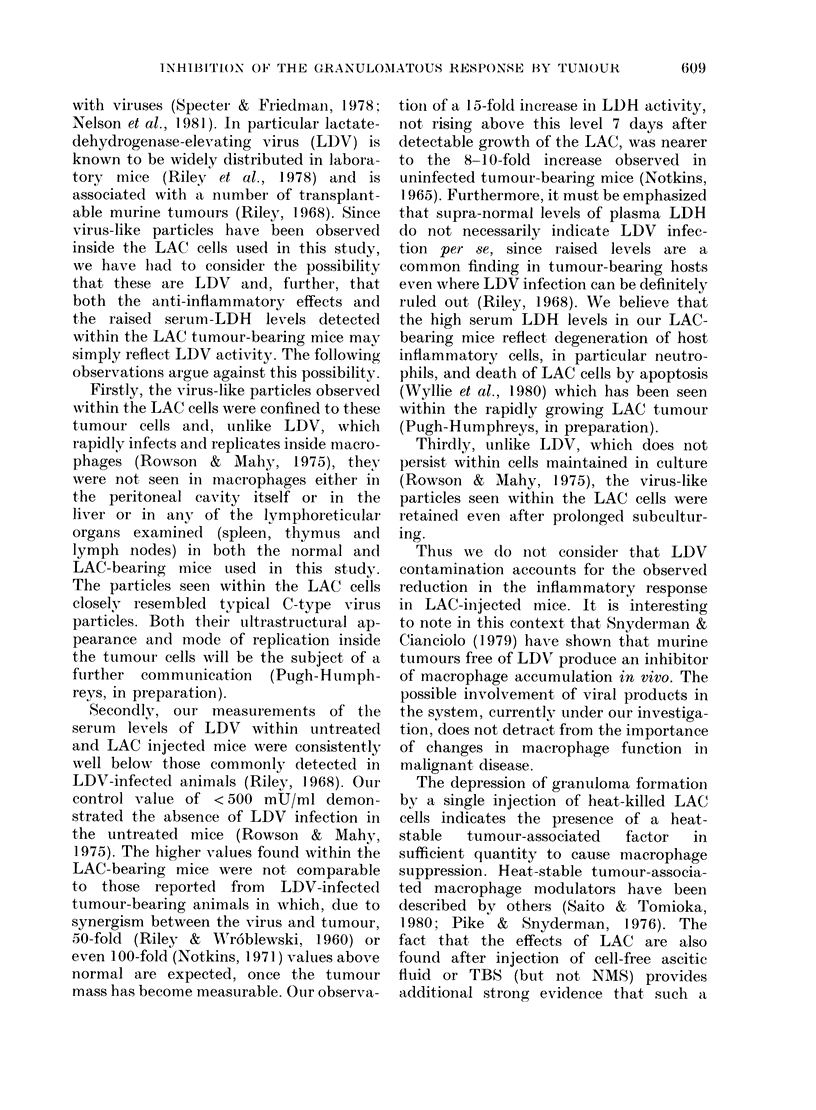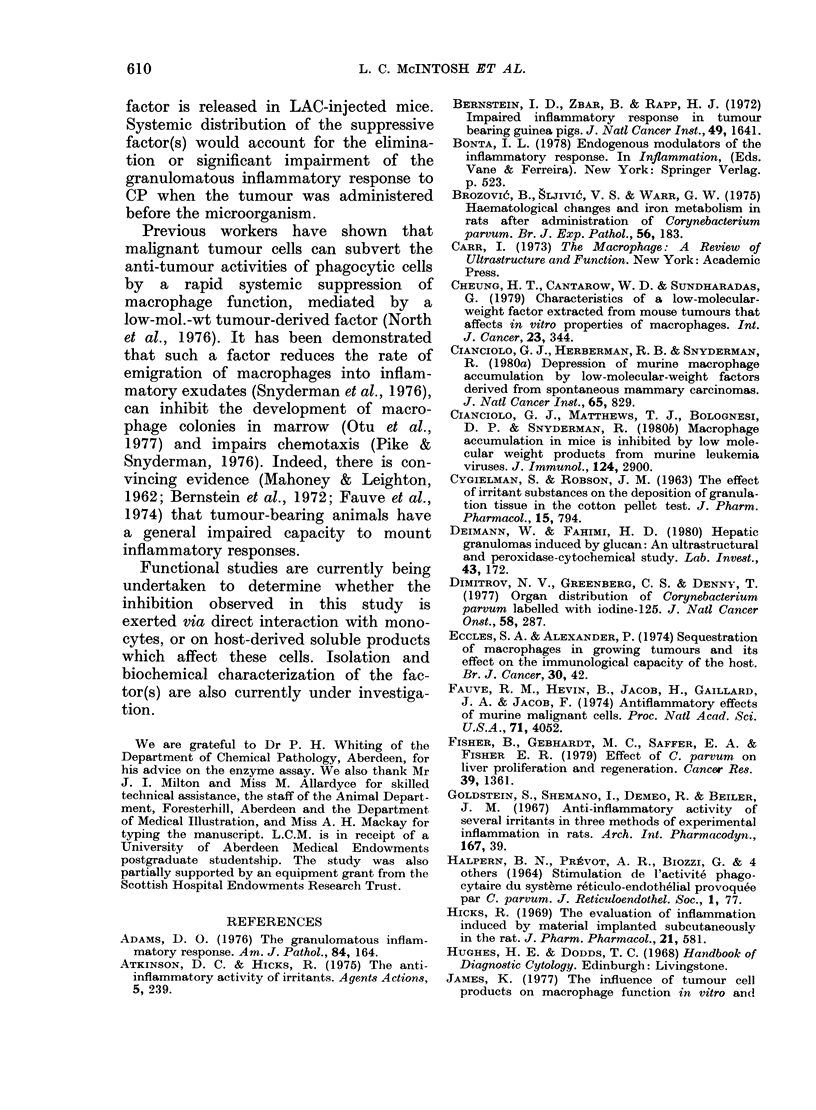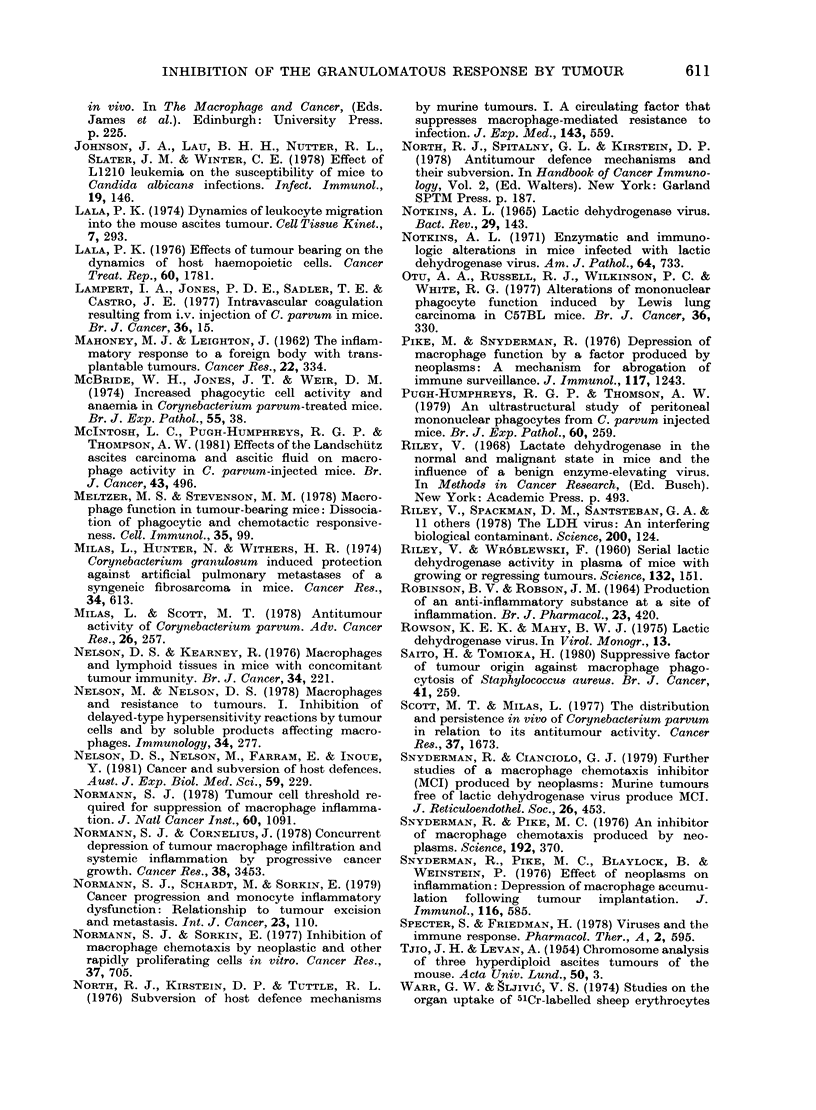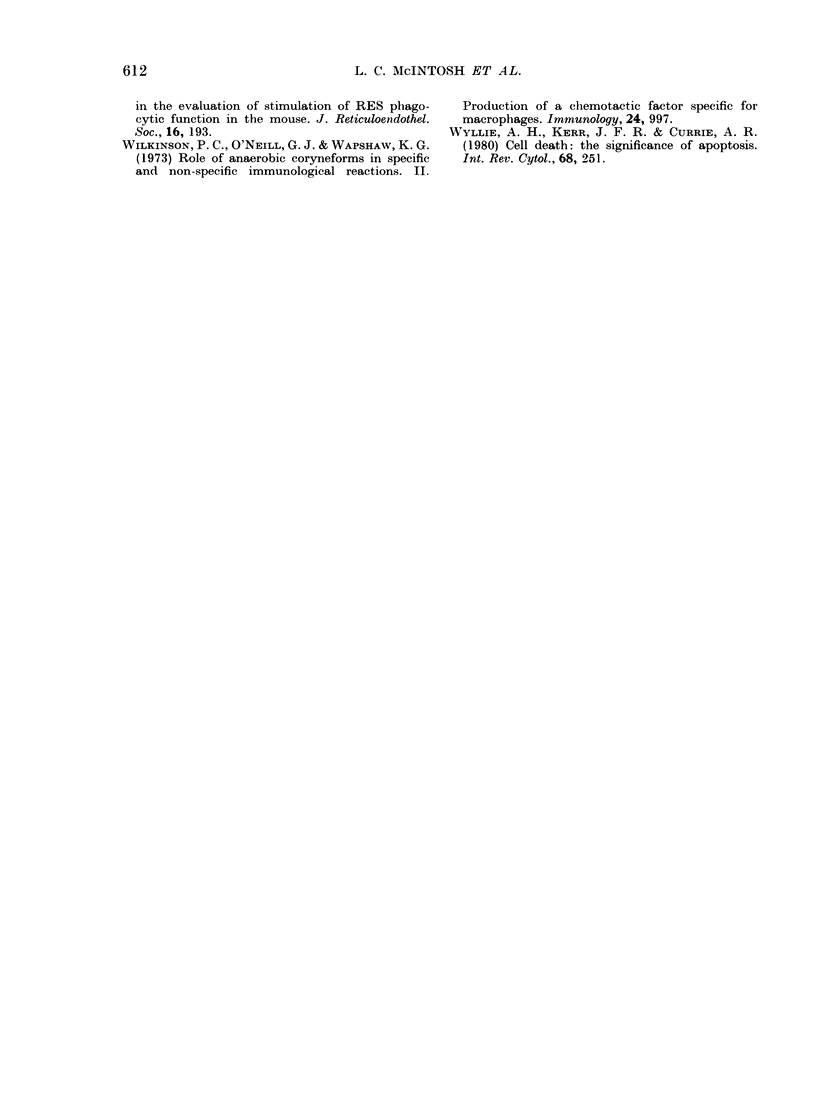# Inhibition by the Landschütz ascites carcinoma of the granulomatous inflammatory response to C. parvum.

**DOI:** 10.1038/bjc.1982.97

**Published:** 1982-04

**Authors:** L. C. McIntosh, R. G. Pugh-Humphreys, R. A. Fraser, A. W. Thomson

## Abstract

**Images:**


Br. J. Cancer (1982) 45, 598

### INHIBITION BY THE LANDSCHÜTZ ASCITES CARCINOMA OF THE GRANULOMATOUS INFLAMMATORY RESPONSE TO *C. PARVUM*

L. C. McINTOSH, R. G. P. PUGH-HUMPHREYS*, R. A. FRASER AND A. W. THOMSON†

*From the Immunopathology Laboratory, Department of Pathology and *Department of Zoology, University of Aberdeen, Aberdeen*

Received 1 September 1981 Accepted 22 December 1981

**Summary.—I.p. or i.v. administration of *Corynebacterium parvum* (CP) to MF1 mice induces a generalized inflammatory response, associated with marked hepatosplenomegaly and accompanied by a pronounced granulomatous response in the liver. Injection of the Landschütz ascites carcinoma (LAC) 24 h after CP substantially reduced the intensity of the inflammatory response, and decreased both the frequency and size of the hepatic granulomas, as revealed by morphometric analysis of histological sections.**

**The difference in cellular composition of the granulomas between the experimental groups, as revealed by light microscopy, was further emphasized and characterized by ultrastructural studies. These revealed the predominance of macrophages within the granulomas in tumour-bearing mice, in contrast to the predominance of epithelioid cells in the lesions which developed in mice given CP alone.**

**Our experimental findings show that the inhibitory effect of the growing LAC on granuloma formation in response to CP cannot be ascribed to (a) sequestration of the microorganism within the growing tumour, (b) a nonspecific inflammatory stimulus, (c) diversion and seqestration of mononuclear phagocytes in the growing tumour or (d) the presence of lactate dehydrogenase-elevating virus in either the host or tumour cells. The inhibition of liver granuloma formation is consistent with an effect mediated by soluble, heat-stable tumour-associated factor(s).**

IT IS WELL RECOGNIZED that systemic administration of *Corynebacterium parvum* (CP) to laboratory animals causes marked stimulation of the mononuclear phagocyte system (MPS), characterized by conspicuous hepatosplenomegaly (Milas & Scott, 1978). This is associated, in the liver, with infiltration of lymphohistiocytic cells (Halpern *et al.*, 1964; Milas *et al.*; 1974; Brozović *et al.*, 1975) which either form granulomas or diffusely infiltrate liver parenchyma (McBride *et al.*, 1974: Milas *et al.*, 1974; Lampert *et al.*, 1977). Hepatomegaly is associated with increased numbers and phagocytic activity of Kupffer cells (Warr & Šljivić, 1974) and in the spleen there is extensive proliferation of macrophages, lymphocytes and haemopoietic cells (Halpern *et al.*, 1964; Brozović *et al.*, 1975).

In a recent study, we found that injection of Landschütz ascites carcinoma (LAC) cells substantially reduced the increase in hepatic phagocytic activity induced by CP (McIntosh *et al.*, 1981). Furthermore, in the same study we found that after injection of the microorganism the incidence of granulomas was markedly reduced in tumour-bearers. We have now conducted further investiga-

† Present address: Kolling Institute of Medical Research, The Royal North Shore Hospital of Sydney, St Leonards, N.S.W., Australia 2065.

tions which reveal that tumour-carriage not only reduces the incidence of CP-induced granulomas, but also arrests the normal sequence of morphological changes within these lesions. We believe that these results provide additional evidence that malignant tumours exert anti-inflammatory effects mediated by humoral factor(s).

### MATERIALS AND METHODS

*Animals.*—Closed-colony-bred female MF1 mice (mean wt 28 g) were used throughout. They were bred in the University Animal Department, Foresterhill, Aberdeen, maintained in a temperature-controlled environment and received Oxoid rat and mouse breeding diet with tap water *ad libitum.*

*Corynebacterium parvum.*—Strain CN 6134 was supplied by Wellcome Research Laboratories, Beckenham, as formalin-killed material (Lot CA 761) at a concentration of 7 mg dry weight washed CP per ml pyrogen-free physiological saline, with 0·01% thiomersal (phenylmercuric nitrate) as preservative. The preparation was stored at 4°C, and mice received 1·4 mg i.p. or 0·35 mg i.v. Controls were injected with an equal volume of Dulbecco "A" phosphate-buffered saline (PBS).

*Tumour.*—The Landschütz ascites carcinoma, a non-strain-specific subline of the Ehrlich diploid carcinoma (Tjio & Levan, 1954) was propagated by i.p. injection of 0·2 ml undiluted cell suspension obtained by peritoneal aspiration on the 7th day of tumour development. The total number of cells injected was $18{\cdot}4 \pm 1{\cdot}8 \times 10^6$ and viability, estimated by trypan-blue dye exclusion, always exceeded 97%.

*Preparation of liver cells.*—Liver tissue from normal MF1 mice was homogenized for 20 min at 37°C in 0·25% v/w pancreatic trypsin (Difco Laboratories, West Molesey) in PBS. The cells were then filtered through stainless-steel gauze, washed in Eagle's minimal essential medium (MEM, Wellcome) and the concentration adjusted to $90 \times 10^6$/ml. Each animal received 0·2 ml i.p.

*Cell-free ascites fluid.*—Peritoneal fluid was collected from ascitic mice 11 days after tumour injection and centrifuged for 30 min at 12,000 *g* (Normann, 1978). The cell-free supernatant was stored at −20°C and thawed just before use. The protein concentration, estimated by the Lowry method was 22·2 mg/ml and mice received 0·5 ml i.p.

*Histology.*—Livers and spleens were fixed in 10% neutral buffered formalin. Paraffin sections were cut at 5 $\mu$m and stained with haematoxylin and eosin (H. & E.). Gram and Twort stains were used to identify CP within the tissues. Mitotic incidence was estimated under a ×10 objective.

*Morphometric analysis of liver granulomas.*—Areas of parenchymal and perivascular (periportal and pericentral vein) granulomas in H. & E. sections of liver were estimated after the method of Deimann & Fahimi (1980). The GDS1 image-analysis system (Graphics Information Systems, Blairgowrie) was used, together with a Leitz Orthoplan light microscope (Leitz Wetzlar, West Germany). Ten randomly selected fields from each section were projected on to a digitizing pad, and the areas of the granulomas were outlined on the graphic tablet which was interfaced to a Tektronix 4051 computer. The results were expressed as ratios of granuloma volume to total liver volume. The latter consisted of the volume of parenchymal and nonparenchymal cells as well as sinusoids, portal triads and central veins, but excluded larger vessels.

*Electron microscopy.*—For transmission electron microscopy (TEM) tissue blocks (1 mm³) were fixed for 4 h at 20°C in 2·5% glutaraldehyde in 0·1M cacodylate buffer, containing 2·5mM $CaCl_2$. After post-fixation in 1% osmium tetroxide the tissue was dehydrated and embedded in TAAB epoxy resin. Ultrathin sections were stained with uranyl acetate and lead citrate before examination in an AEI EM6B transmission electron microscope.

*Morphological characterization of peritoneal cells.*—Peritoneal cavities were lavaged with MEM containing 2i.u. heparin (Duncan, Flockhart and Co., London) per ml. One-ml aliquots of washed cells from each of 5 mice were pooled, and replicate counts (each based on at least 200 cells) of the different cell types were made on Romanowsky-stained (Haema-Tek system; Miles Laboratories, Slough) cytospin preparations. The tumour cells were identified as large mononuclear (occasionally multinucleate) forms, with a large eccentric nucleus with prominent dense clumps of chromatin. The other characteristic feature (Hughes & Dodds, 1968)

was a prominent pale-staining Golgi region adjacent to the nucleus and associated with numerous granules and vacuoles.

*Assay for LDH*.—Individual blood samples were collected from normal and tumour-bearing mice by cardiac puncture, and the serum obtained was then assayed for lactate dehydrogenase (LDH) activity. The pyruvate-dependent oxidation of reduced nicotinamide-adenine dinucleotide was measured at 27°C at 340 nm on a recording UV spectrophotometer, essentially by the method of Rowson & Mahy (1975). The serum LDH activity was calculated by

$$\text{LDH (mU/ml)} = \frac{\Delta \text{E/min} \times 3 \times 10^6}{6{\cdot}22 \times 10^3 \times \text{ml sample used}}$$

*Statisctics*.—The significance of differences between means was established using a 2-tailed $t$ test for unpaired samples.

### RESULTS

Injection of viable tumour 24 h after CP significantly suppressed ($P < 0{\cdot}005$)

FIG. 1.—(a) Liver and (b) spleen weights of mice 8 days after i.p. *C. parvum*. The additional injections were given i.p. 24 h after the microorganism. Also shown are organ weights in mice given tumour alone. Results are means ±s.d. for 4–9 mice. HK tumour = heat-killed ($18 \times 10^6$ cells heated at 56° for 30 min); Tumour fluid = 0·5 ml 11-day cell-free ascitic fluid; TBS = 0·5 ml pooled 11-day tumour-bearer serum; NMS = 0·5 ml pooled normal mouse serum.

the marked hepatosplenomegaly evident 8 days after i.p. administration of the microorganism (Fig. 1). No such inhibition was seen in animals given heat-killed tumour-cell suspension, ascitic fluid, tumour-bearer (TBS) or normal mouse serum (NMS). Indeed, ascitic fluid and NMS caused a small but significant increase ($P < 0{\cdot}05$) in liver weight over that produced by CP.

Morphometric analysis of the livers revealed that in tumour-bearing animals there was a very striking reduction in the degree of granuloma formation in response to CP (Fig. 2). In mice given CP alone, about 15% of the total parenchymal volume at 8 days was occupied by granuloma tissue, of which 24% was perivascular granuloma. The injection of viable or heat-killed tumour, ascitic fluid

FIG. 2.—Volume of liver granulomas 8 days after i.p. *C. parvum*, estimated by image analysis. The additional injections were given i.p. 24 h after the microorganism. Results are means ±s.d. for 4–6 mice. Abbreviations as in Fig. 1.

or TBS significantly depressed ($P < 0{\cdot}005$) both parenchymal and perivascular granuloma production. No significant effect was obtained with NMS. A striking change was also noted in the morphological characteristics of the granulomas in the tumour-bearers; instead of containing "epithelioid" cells as seen in response to CP alone, they contained predominantly mononuclear cells (Fig. 3).

TEM revealed that those liver granulomas which developed in the mice injected with CP and tumour cells contained small monocytes 10–14 $\mu$m in diameter, which resembled circulating blood monocytes, and larger cells 14–20 $\mu$m in diameter, with ultrastructural features resembling those of tissue monocytes (see Carr, 1973). Although both types of cells contained mitochondria, endoplasmic reticulum, lysosomes and Golgi apparatus, there was hypertrophy of the Golgi apparatus within the larger tissue monocytes and an increase in the number of lysosomes. In those granulomas developing within the mice injected with CP alone, there were epithelioid cells in addition to monocytic cells. The epithelioid cells contained mitochondria, 5 nm-diameter microfilaments, active Golgi apparatus, endoplasmic reticulum, lysosomes and vacuoles containing flocculates of moderately electron-dense material. The plasma membranes of the epithelioid cells were thrown into numerous villiform processes, and there was extensive interdigitation of adjacent epithelioid-cell plasma membranes, forming a reticular pattern (Fig. 4). However, no structures resembling desmosomes were found between the contiguous membranes of adjacent epithelioid cells.

Examination of Gram- and Twort-stained sections of liver and spleen revealed that the tumour did not affect the uptake or distribution of CP within mononuclear phagocytes. However, the tumour did cause a marked reduction ($\sim 60\%$) in the increased incidence of mitotic figures within the liver parenchyma in response to CP. This inhibition was reduced with heat-killed tumour, ascitic fluid and TBS, but was not seen in response to NMS.

Additional studies were conducted to examine the specificity of this apparent inhibition by LAC of the granulomatous response to CP. In order to establish that the tumour was not acting simply as a diversionary stimulus for cells which might otherwise populate the liver in response to CP, absolute and differential counts of host cells within the peritoneum were made. It is clear from these results (Fig. 5) that injection of LAC 24 h after CP caused no further significant increase in the absolute numbers of lymphocytes, macrophages or granulocytes. Indeed, macrophage numbers were quite strikingly depressed after tumour injection, especially in the CP group. In the spleen, the pronounced increase in macrophage

FIG. 3.—Liver sections (a) 8 days after i.p. CP, showing pronounced parenchymal and perivascular granulomas; (b) shows their epithelioid nature; (c) 8 days after CP and 7 days after tumour, showing reduction in granuloma volume and (d) their "lymphohistiocytic" nature. All H. & E.; (a) and (c) × 120, (b) and (d) × 300.

FIG. 4.—(a) Electron micrograph showing epithelioid cells within a CP-induced granuloma. The plasma membranes of the epithelioid cells are thrown into slender villiform processes, and there is characteristic extensive interdigitation of the plasma membranes of adjacent cells ( × 9500). (b) Electron micrograph of granuloma in mouse given CP 8 days and tumour 7 days previously, showing a small monocyte and the larger tissue monocyte. Whereas both cells contain lysosomes, the latter cell contains a more active Golgi apparatus ( × 6000). (c) The predominant cells within the granuloma are tissue macrophages containing prominent lysosomes. ( × 9500).

FIG. 5.—Absolute lymphocyte (●), monocyte (■) and granulocyte (◇) counts within the peritoneal cavities at various times after injection of tumour, CP + tumour and CP alone. Each point represents the result from 5 animals.

numbers seen in the marginal zones in response to CP was absent from mice which also received tumour (Fig. 6).

The inhibitory effects of i.p. tumour given 24 h after CP, were also seen in mice given the microorganism by the i.v. route (Table I). In addition to inhibition of CP-induced hepatosplenomegaly, overall granuloma production was reduced by 87%, with a more marked effect on parenchymal than perivascular granulomas. Injection of the tumour 3 days before CP (i.p. or i.v.) had similar effects on organ weights, and either totally eliminated the granulomatous response (i.p. injection) or reduced it by 57% (i.v. route) (Table II).

A potent inflammatory stimulus (sodium caseinate) given once by the i.p. route 24 h after CP did not affect the increase in organ weights, but did significantly inhibit (by 38%) granuloma production (Table III). This effect was considerably less than the 87% inhibition induced by tumour under similar conditions (see Fig. 2 and Table I) and was not significantly enhanced by repeated injection of the caseinate. Moreover, sodium caseinate did not impair the progression to "epithelioid" granulomas.

Injection of an equal number of viable normal liver cells did not, in contrast to tumour cells, significantly reduce either CP-induced hepatosplenomegaly or granuloma production (Table IV).

There was no significant difference between the plasma and serum levels of LDH activity found in control animals, so the levels in experimental animals were expressed in terms of serum activity (Table V). A 5-fold increase in this level was obtained 2 days after injection of viable tumour, rising to 15-fold by Day 4 (when the tumour mass becomes measurable). This level was maintained over the next 7 days (*i.e.* Day 11 not significantly different from Day 4, though Day 7 was lower, $P < 0{\cdot}01$).

### DISCUSSION

There is substantial evidence that the various activities of the MPS are affected during the course of tumour growth in experimental animals (James, 1977; North *et al.*, 1978; Nelson *et al.*, 1981). Whereas in some instances tumours appear to have an enhancing effect on MPS responses (Nelson & Kearney, 1976; Meltzer & Stevenson, 1978), in other cases a depressive effect has been reported (Eccles & Alexander, 1974; Pike & Snyderman, 1976; Nelson & Nelson, 1978; Johnson *et al.*, 1978; Normann & Cornelius, 1978;

(a)

(b)

FIG. 6.—Spleen (a) 8 days after i.p. CP, showing pronounced increase in marginal zone macrophages; (b) 8 days after CP and 7 days after tumour, showing absence of this response H. & E. ×120.

TABLE I.—*Effect of tumour on granuloma production induced by i.v.* C. parvum

| Treatment | Organ weight (g/25 g body wt) Liver | Spleen | Volume of granulomas (% liver tissue) Parenchymal | Perivascular |
|---|---|---|---|---|
| Control (normal) | 1·45±0·11 | 0·09±0·02 | 0 | 0 |
| Tumour | 1·78±0·24 | 0·21±0·08** | 0 | 0 |
| CP | 2·37±0·32 | 0·29±0·10 | 7·51±2·30 | 2·78±0·78 |
| CP+tumour (Day 1) | 1·80±0·22* | 0·19±0·06 | 0·68±0·44** | 0·68±0·44** |

Results are means ±s.d. for 4–6 mice, 8 days after CP injection. Tumour was injected i.p. 24 h after CP.
Asterisks indicate significance of difference from corresponding (adjacent) controls: * $P<0·02$; ** $P<0·005$.

TABLE II.—*Effect of tumour injection before CP on granuloma production*

| Treatment | Organ weight (g/25 g body wt) Liver | Spleen | Volume of granulomas (% liver tissue) Parenchymal | Perivascular |
|---|---|---|---|---|
| Control | 1·45±0·14 | 0·12±0·02 | 0 | 0 |
| Tumour (Day−3) | 1·95±0·28 | 0·11±0·03 | 0 | 0 |
| CP (i.p.) | 2·52±0·22 | 0·44±0·07 | 11·85±1·75 | 3·63±0·75 |
| Tumour (Day−3)+CP (i.p.) | 1·11±0·10** | 0·09±0·02** | 0 | 0 |
| CP (i.v.) | 2·37±0·32 | 0·29±0·10 | 7·51±2·30 | 2·78±0·78 |
| Tumour (Day−3)+CP (i.v.) | 1·91±0·22* | 0·21±0·06 | 3·23±2·51* | 1·78±0·78 |

*C. parvum* injected i.p. or i.v. on Day 0.
Results are means ±s.d. on Day 8.
Asterisks indicate significance of difference from corresponding (adjacent) controls: * $P<0·02$; ** $P<0·005$.

TABLE III.—*Effect of an inflammatory stimulus (sodium caseinate) on CP-induced granuloma production*

| Treatment | Organ weight (g/25 g body wt) Liver | Spleen | Volume of granulomas (% liver tissue) Parenchymal | Perivascular |
|---|---|---|---|---|
| Normal | 1·71±0·31 | 0·12±0·04 | 0 | 0 |
| CP+saline (Day 1) | 2·52±0·22 | 0·44±0·07 | 11·84±1·80 | 3·67±0·58 |
| CP+Na cas. (Day 1) | 2·20±0·20 | 0·40±0·06 | 7·64±1·04** | 2·03±0·45** |
| CP+saline (Days 1, 3, 5) | 3·03±0·55 | 0·42±0·10 | 8·81±4·77 | 3·82±1·02 |
| CP+Na cas. (Days 1, 3, 5) | 2·64±0·23 | 0·32±0·11 | 4·89±2·53 | 1·49±1·05* |

Values are means±s.d. on Day 8.
Na cas. = sodium caseinate (0·5 ml 3·5% in PBS i.p.).
CP injected i.p.
Asterisks indicate significance of difference from corresponding (adjacent) controls: * $P<0·01$; ** $P<0·005$.

TABLE IV.—*Effect of liver cell injection on CP-induced granuloma production*

| Treatment | Organ weight (g/25 g body wt) Liver | Spleen | Volume of granulomas (% liver tissue) Parenchymal | Perivascular |
|---|---|---|---|---|
| Control | 1·71±0·31 | 0·12±0·04 | 0 | 0 |
| Liver cells | 1·81±0·15 | 0·14±0·02 | 0 | 0 |
| CP | 2·52±0·22 | 0·44±0·07 | 11·84±1·80 | 3·67±0·58 |
| CP+liver cells | 3·00±0·44* | 0·37±0·06 | 9·63±2·28 | 2·78±1·03 |

CP given i.p.
Values are means±s.d. for 4–6 mice on Day 8.
Liver cells were injected i.p. 24 h after CP.
Asterisk indicates significance of difference from corresponding (adjacent) control: * $P<0·05$.

TABLE V.—*Effect of tumour on serum LDH activity*

| Treatment | LDH activity (mU/ml) |
|---|---|
| Control (normal) | 309 ± 119* |
| Tumour (Day 2) | 1685 ± 407 |
| (Day 4) | 4097 ± 1410 |
| (Day 7) | 2823 ± 247 |
| (Day 11) | 5116 ± 858 |

Values are means ± 1 s.d. obtained from groups of 6–10 mice.
* Plasma level: 374 ± 392.

Normann *et al.*, 1979; Cheung *et al.*, 1979; Cianciolo *et al.*, 1980*a*, *b*). In this study, we substantiate our previous finding (McIntosh *et al.*, 1981) that the granulomatous inflammatory response to CP, is impaired by injection of LAC cells.

Systemic CP is associated with hepatosplenomegaly and the infiltration of inflammatory cells into a number of organs, including the liver and spleen (Milas & Scott, 1978). These tissues then show a characteristic granulomatous response (see *e.g.* Adams, 1976) with the transformation of recruited blood monocytes into tissue monocytes and epithelioid cells. This response was confirmed in the present study by the development of lymphohistiocytic and epithelioid granulomas 5 days after i.p. CP injection; only the latter type were evident by Day 8. No giant cells were found, in contrast to the observation of Brozović *et al.* (1975) that giant cells of the Langhans type appeared in addition to epithelioid cells within CP-induced granulomas in rat liver and spleen.

Injection of tumour 24 h after the microorganism produced both a decrease in the frequency and size of granulomas and a significant difference in their cellular composition. In tumour-bearers, the lymphohistiocytic lesions observed at Day 8 resembled those seen at an earlier stage (Day 5) in animals given CP alone. Our results suggest that the presence of tumour alters the usual progression of the granulomatous response to CP and imply impairment of both cell recruitment and transformation of histiocytes to epithelioid cells.

In CP-treated mice bearing tumour, a decrease in liver and spleen weight was also found. Hepatosplenomegaly is determined by the amount of CP entering the tissues (Scott & Milas, 1977). Although it is conceivable that some of the CP administered by the i.p. route could have been sequestered within i.p. tumour given 24 h after the microorganism, the LAC cells are not phagocytic, and we have never seen CP within them. Furthermore, 24 h is considered to be sufficient time for i.p. CP to accumulate in the liver and spleen. When total organ recovery rates for $^{125}$I-labelled CP given i.p. or i.v. were compared (Scott & Milas, 1977; Dimitrov *et al.*, 1977) it was found that the highest recovery rates were obtained from i.v. CP. These findings imply different processing of CP after i.p. and i.v. injection. It is possible that peritoneal macrophages could modify the processing of CP after i.p. administration (Dimitrov *et al.*, 1977) since these cells do take up the microorganism (Pugh-Humphreys & Thomson, 1979; Scott & Milas, 1977). Therefore, i.v. injection greatly reduces the possibility of CP being sequestered within the site of tumour growth, due to lack of direct contact with cells in the peritoneum. Our arguments are further strengthened therefore by similar results obtained with i.v. CP. Thus the decrease in hepatosplenomegaly in response to i.v. CP given 24 h before tumour can hardly be explained in terms of lack of availability of the microorganism. Indeed, 5 days after its i.p. administration CP could easily be seen within livers of tumour-bearing mice.

The characteristic hepatosplenomegaly was decreased only by the presence of viable tumour. Other agents (heat-killed LAC, ascitic fluid, TBS) whilst causing a similar decrease in the inflammatory response in these organs, did not affect organ weights. It has been found (Fisher *et al.*, 1979) that the increase in weight of rat livers is attributable not only to the lymphohistiocytic infiltrate which occurs in response to the microorganism, but also to hepatocyte proliferation. However, in our study, histological examination of those livers showing a reduced granulomatous response also revealed a decrease in parenchymal mitosis. Thus the weight changes are not directly related to the extent of granuloma formation or hepatocyte proliferation, but could be due to increased water content or an increased presence of intracellular hepatic fat or glycogen (Fisher *et al.*, 1979).

A strong chemotactic factor is associated with CP (Wilkinson *et al.*, 1973) and mobilizes mononuclear cells into the liver and spleen. Although in the present study CP was seen in phagocytic cells within livers and spleens of both normal and tumour-bearing mice, there was a marked reduction in the intensity of the mononuclear-cell infiltrates in animals bearing LAC. The decrease in hepatic and splenic infiltrates could be accounted for by a diversionary effect of i.p. tumour on cell migration into these organs, to decreased production of these cells in the marrow, to impairment of chemotaxis, or to a combination of these.

A number of different types of host cell invade the Ehrlich ascites tumour (EAT) (Lala, 1974) of which the LAC is a subline. These include mononuclear cells selectively recruited from the blood and ultimately derived largely from the marrow (Lala, 1974, 1976). From our data it is clear that during the first 3 days of tumour growth there was a depression of absolute monocyte numbers within the tumour; these were gradually restored to normal from Day 4 onwards. In mice given CP alone, there was only a very short, transient depression, followed by a rapid rise. In contrast, CP-treated mice given tumour showed a sustained depression of monocyte infiltration into the tumour. This effect coincided with decreased monocyte infiltration into the livers and spleens of these animals. It is hardly likely, therefore, that the tumour acts simply by diverting inflammatory cells which would otherwise have colonized the liver and spleen. In tumour-bearing mice, PMN infiltration was unaffected in accordance with the findings of others (Snyderman & Pike, 1976; Normann & Sorkin, 1977) that, whilst tumours inhibit macrophage accumulation *in vivo*, they have little such effect on PMN leucocytes.

Our data do not allow us to ascertain whether impairment of chemotaxis or monocytopoiesis cause the observed anti-inflammatory effect of the tumour. Indeed, both mechanisms could be acting. We nevertheless favour the latter explanation, since there were no large accumulations of monocytes in the three major lymphoreticular sites examined and, furthermore, additional investigations (to be reported) have not revealed substantial numbers of these cells in the circulation.

Granuloma formation has been shown to be sensitive to "counter-irritation" (Cygielman & Robson, 1963; Goldstein *et al.*, 1967; Hicks, 1969; Robinson & Robson, 1964,) the process whereby inflammation due to an inert irritant at one site results in the production not only of inflammatory mediators but also endogenous (host-derived) anti-inflammatory factors which are carried *via* the blood stream and can suppress inflammation leading to granuloma formation at another site (Atkinson & Hicks, 1975; Bonta, 1978). The failure of single or repeated injections of a potent inflammatory stimulus (sodium caseinate) to depress granuloma formation within the livers comparable to that of the LAC further substantiates our view that the tumour is not acting as a nonspecific inflammatory stimulus, and thus is not acting simply as a "counter-irritant". Since the injection of normal viable mouse liver cells did not affect the granulomatous response to CP the effect of tumour is presumably specific, and not simply due to the introduction of viable cells into the peritoneum.

Many abnormalities in immune function, including suppression in macrophage function, occur in mice infected with viruses (Specter & Friedman, 1978; Nelson *et al.*, 1981). In particular lactate-dehydrogenase-elevating virus (LDV) is known to be widely distributed in laboratory mice (Riley *et al.*, 1978) and is associated with a number of transplantable murine tumours (Riley, 1968). Since virus-like particles have been observed inside the LAC cells used in this study, we have had to consider the possibility that these are LDV and, further, that both the anti-inflammatory effects and the raised serum-LDH levels detected within the LAC tumour-bearing mice may simply reflect LDV activity. The following observations argue against this possibility.

Firstly, the virus-like particles observed within the LAC cells were confined to these tumour cells and, unlike LDV, which rapidly infects and replicates inside macrophages (Rowson & Mahy, 1975), they were not seen in macrophages either in the peritoneal cavity itself or in the liver or in any of the lymphoreticular organs examined (spleen, thymus and lymph nodes) in both the normal and LAC-bearing mice used in this study. The particles seen within the LAC cells closely resembled typical C-type virus particles. Both their ultrastructural appearance and mode of replication inside the tumour cells will be the subject of a further communication (Pugh-Humphreys, in preparation).

Secondly, our measurements of the serum levels of LDV within untreated and LAC injected mice were consistently well below those commonly detected in LDV-infected animals (Riley, 1968). Our control value of $<500$ mU/ml demonstrated the absence of LDV infection in the untreated mice (Rowson & Mahy, 1975). The higher values found within the LAC-bearing mice were not comparable to those reported from LDV-infected tumour-bearing animals in which, due to synergism between the virus and tumour, 50-fold (Riley & Wróblewski, 1960) or even 100-fold (Notkins, 1971) values above normal are expected, once the tumour mass has become measurable. Our observation of a 15-fold increase in LDH activity, not rising above this level 7 days after detectable growth of the LAC, was nearer to the 8–10-fold increase observed in uninfected tumour-bearing mice (Notkins, 1965). Furthermore, it must be emphasized that supra-normal levels of plasma LDH do not necessarily indicate LDV infection *per se*, since raised levels are a common finding in tumour-bearing hosts even where LDV infection can be definitely ruled out (Riley, 1968). We believe that the high serum LDH levels in our LAC-bearing mice reflect degeneration of host inflammatory cells, in particular neutrophils, and death of LAC cells by apoptosis (Wyllie *et al.*, 1980) which has been seen within the rapidly growing LAC tumour (Pugh-Humphreys, in preparation).

Thirdly, unlike LDV, which does not persist within cells maintained in culture (Rowson & Mahy, 1975), the virus-like particles seen within the LAC cells were retained even after prolonged subculturing.

Thus we do not consider that LDV contamination accounts for the observed reduction in the inflammatory response in LAC-injected mice. It is interesting to note in this context that Snyderman & Cianciolo (1979) have shown that murine tumours free of LDV produce an inhibitor of macrophage accumulation *in vivo*. The possible involvement of viral products in the system, currently under our investigation, does not detract from the importance of changes in macrophage function in malignant disease.

The depression of granuloma formation by a single injection of heat-killed LAC cells indicates the presence of a heat-stable tumour-associated factor in sufficient quantity to cause macrophage suppression. Heat-stable tumour-associated macrophage modulators have been described by others (Saito & Tomioka, 1980; Pike & Snyderman, 1976). The fact that the effects of LAC are also found after injection of cell-free ascitic fluid or TBS (but not NMS) provides additional strong evidence that such a factor is released in LAC-injected mice. Systemic distribution of the suppressive factor(s) would account for the elimination or significant impairment of the granulomatous inflammatory response to CP when the tumour was administered before the microorganism.

Previous workers have shown that malignant tumour cells can subvert the anti-tumour activities of phagocytic cells by a rapid systemic suppression of macrophage function, mediated by a low-mol.-wt tumour-derived factor (North *et al.*, 1976). It has been demonstrated that such a factor reduces the rate of emigration of macrophages into inflammatory exudates (Snyderman *et al.*, 1976), can inhibit the development of macrophage colonies in marrow (Otu *et al.*, 1977) and impairs chemotaxis (Pike & Snyderman, 1976). Indeed, there is convincing evidence (Mahoney & Leighton, 1962; Bernstein *et al.*, 1972; Fauve *et al.*, 1974) that tumour-bearing animals have a general impaired capacity to mount inflammatory responses.

Functional studies are currently being undertaken to determine whether the inhibition observed in this study is exerted *via* direct interaction with monocytes, or on host-derived soluble products which affect these cells. Isolation and biochemical characterization of the factor(s) are also currently under investigation.

We are grateful to Dr P. H. Whiting of the Department of Chemical Pathology, Aberdeen, for his advice on the enzyme assay. We also thank Mr J. I. Milton and Miss M. Allardyce for skilled technical assistance, the staff of the Animal Department, Foresterhill, Aberdeen and the Department of Medical Illustration, and Miss A. H. Mackay for typing the manuscript. L.C.M. is in receipt of a University of Aberdeen Medical Endowments postgraduate studentship. The study was also partially supported by an equipment grant from the Scottish Hospital Endowments Research Trust.

### REFERENCES

ADAMS, D. O. (1976) The granulomatous inflammatory response. *Am. J. Pathol.*, **84**, 164.

ATKINSON, D. C. & HICKS, R. (1975) The anti-inflammatory activity of irritants. *Agents Actions*, **5**, 239.

BERNSTEIN, I. D., ZBAR, B. & RAPP, H. J. (1972) Impaired inflammatory response in tumour bearing guinea pigs. *J. Natl Cancer Inst.*, **49**, 1641.

BONTA, I. L. (1978) Endogenous modulators of the inflammatory response. In *Inflammation*, (Eds. Vane & Ferreira). New York: Springer Verlag. p. 523.

BROZOVIĆ, B., ŠLJIVIĆ, V. S. & WARR, G. W. (1975) Haematological changes and iron metabolism in rats after administration of *Corynebacterium parvum*. *Br. J. Exp. Pathol.*, **56**, 183.

CARR, I. (1973) *The Macrophage: A Review of Ultrastructure and Function.* New York: Academic Press.

CHEUNG, H. T., CANTAROW, W. D. & SUNDHARADAS, G. (1979) Characteristics of a low-molecular-weight factor extracted from mouse tumours that affects *in vitro* properties of macrophages. *Int. J. Cancer*, **23**, 344.

CIANCIOLO, G. J., HERBERMAN, R. B. & SNYDERMAN, R. (1980*a*) Depression of murine macrophage accumulation by low-molecular-weight factors derived from spontaneous mammary carcinomas. *J. Natl Cancer Inst.*, **65**, 829.

CIANCIOLO, G. J., MATTHEWS, T. J., BOLOGNESI, D. P. & SNYDERMAN, R. (1980*b*) Macrophage accumulation in mice is inhibited by low molecular weight products from murine leukemia viruses. *J. Immunol.*, **124**, 2900.

CYGIELMAN, S. & ROBSON, J. M. (1963) The effect of irritant substances on the deposition of granulation tissue in the cotton pellet test. *J. Pharm. Pharmacol.*, **15**, 794.

DEIMANN, W. & FAHIMI, H. D. (1980) Hepatic granulomas induced by glucan: An ultrastructural and peroxidase-cytochemical study. *Lab. Invest.*, **43**, 172.

DIMITROV, N. V., GREENBERG, C. S. & DENNY, T. (1977) Organ distribution of *Corynebacterium parvum* labelled with iodine-125. *J. Natl Cancer Onst.*, **58**, 287.

ECCLES, S. A. & ALEXANDER, P. (1974) Sequestration of macrophages in growing tumours and its effect on the immunological capacity of the host. *Br. J. Cancer*, **30**, 42.

FAUVE, R. M., HEVIN, B., JACOB, H., GAILLARD, J. A. & JACOB, F. (1974) Antiflammatory effects of murine malignant cells. *Proc. Natl Acad. Sci. U.S.A.*, **71**, 4052.

FISHER, B., GEBHARDT, M. C., SAFFER, E. A. & FISHER E. R. (1979) Effect of *C. parvum* on liver proliferation and regeneration. *Cancer Res.* **39**, 1361.

GOLDSTEIN, S., SHEMANO, I., DEMEO, R. & BEILER, J. M. (1967) Anti-inflammatory activity of several irritants in three methods of experimental inflammation in rats. *Arch. Int. Pharmacodyn.*, **167**, 39.

HALPERN, B. N., PRÉVOT, A. R., BIOZZI, G. & 4 others (1964) Stimulation de l'activité phagocytaire du système réticulo-endothélial provoquée par *C. parvum*. *J. Reticuloendothel. Soc.*, **1**, 77.

HICKS, R. (1969) The evaluation of inflammation induced by material implanted subcutaneously in the rat. *J. Pharm. Pharmacol.*, **21**, 581.

HUGHES, H. E. & DODDS, T. C. (1968) *Handbook of Diagnostic Cytology.* Edinburgh: Livingstone.

JAMES, K. (1977) The influence of tumour cell products on macrophage function *in vitro* and

*in vivo*. In *The Macrophage and Cancer*, (Eds. James *et al*.). Edinburgh: University Press. p. 225.

JOHNSON, J. A., LAU, B. H. H., NUTTER, R. L., SLATER, J. M. & WINTER, C. E. (1978) Effect of L1210 leukemia on the susceptibility of mice to *Candida albicans* infections. *Infect. Immunol.*, **19**, 146.

LALA, P. K. (1974) Dynamics of leukocyte migration into the mouse ascites tumour. *Cell Tissue Kinet.*, **7**, 293.

LALA, P. K. (1976) Effects of tumour bearing on the dynamics of host haemopoietic cells. *Cancer Treat. Rep.*, **60**, 1781.

LAMPERT, I. A., JONES, P. D. E., SADLER, T. E. & CASTRO, J. E. (1977) Intravascular coagulation resulting from i.v. injection of *C. parvum* in mice. *Br. J. Cancer*, **36**, 15.

MAHONEY, M. J. & LEIGHTON, J. (1962) The inflammatory response to a foreign body with transplantable tumours. *Cancer Res.*, **22**, 334.

MCBRIDE, W. H., JONES, J. T. & WEIR, D. M. (1974) Increased phagocytic cell activity and anaemia in *Corynebacterium parvum*-treated mice. *Br. J. Exp. Pathol.*, **55**, 38.

MCINTOSH, L. C., PUGH-HUMPHREYS, R. G. P. & THOMPSON, A. W. (1981) Effects of the Landschütz ascites carcinoma and ascitic fluid on macrophage activity in *C. parvum*-injected mice. *Br. J. Cancer*, **43**, 496.

MELTZER, M. S. & STEVENSON, M. M. (1978) Macrophage function in tumour-bearing mice: Dissociation of phagocytic and chemotactic responsiveness. *Cell. Immunol.*, **35**, 99.

MILAS, L., HUNTER, N. & WITHERS, H. R. (1974) *Corynebacterium granulosum* induced protection against artificial pulmonary metastases of a syngeneic fibrosarcoma in mice. *Cancer Res.*, **34**, 613.

MILAS, L. & SCOTT, M. T. (1978) Antitumour activity of *Corynebacterium parvum*. *Adv. Cancer Res.*, **26**, 257.

NELSON, D. S. & KEARNEY, R. (1976) Macrophages and lymphoid tissues in mice with concomitant tumour immunity. *Br. J. Cancer*, **34**, 221.

NELSON, M. & NELSON, D. S. (1978) Macrophages and resistance to tumours. I. Inhibition of delayed-type hypersensitivity reactions by tumour cells and by soluble products affecting macrophages. *Immunology*, **34**, 277.

NELSON, D. S., NELSON, M., FARRAM, E. & INOUE, Y. (1981) Cancer and subversion of host defences. *Aust. J. Exp. Biol. Med. Sci.*, **59**, 229.

NORMANN, S. J. (1978) Tumour cell threshold required for suppression of macrophage inflammation. *J. Natl Cancer Inst.*, **60**, 1091.

NORMANN, S. J. & CORNELIUS, J. (1978) Concurrent depression of tumour macrophage infiltration and systemic inflammation by progressive cancer growth. *Cancer Res.*, **38**, 3453.

NORMANN, S. J., SCHARDT, M. & SORKIN, E. (1979) Cancer progression and monocyte inflammatory dysfunction: Relationship to tumour excision and metastasis. *Int. J. Cancer*, **23**, 110.

NORMANN, S. J. & SORKIN, E. (1977) Inhibition of macrophage chemotaxis by neoplastic and other rapidly proliferating cells *in vitro*. *Cancer Res.*, **37**, 705.

NORTH, R. J., KIRSTEIN, D. P. & TUTTLE, R. L. (1976) Subversion of host defence mechanisms by murine tumours. I. A circulating factor that suppresses macrophage-mediated resistance to infection. *J. Exp. Med.*, **143**, 559.

NORTH, R. J., SPITALNY, G. L. & KIRSTEIN, D. P. (1978) Antitumour defence mechanisms and their subversion. In *Handbook of Cancer Immunology*, Vol. 2, (Ed. Walters). New York: Garland SPTM Press. p. 187.

NOTKINS, A. L. (1965) Lactic dehydrogenase virus. *Bact. Rev.*, **29**, 143.

NOTKINS, A. L. (1971) Enzymatic and immunologic alterations in mice infected with lactic dehydrogenase virus. *Am. J. Pathol.*, **64**, 733.

OTU, A. A., RUSSELL, R. J., WILKINSON, P. C. & WHITE, R. G. (1977) Alterations of mononuclear phagocyte function induced by Lewis lung carcinoma in C57BL mice. *Br. J. Cancer*, **36**, 330.

PIKE, M. & SNYDERMAN, R. (1976) Depression of macrophage function by a factor produced by neoplasms: A mechanism for abrogation of immune surveillance. *J. Immunol.*, **117**, 1243.

PUGH-HUMPHREYS, R. G. P. & THOMSON, A. W. (1979) An ultrastructural study of peritoneal mononuclear phagocytes from *C. parvum* injected mice. *Br. J. Exp. Pathol.*, **60**, 259.

RILEY, V. (1968) Lactate dehydrogenase in the normal and malignant state in mice and the influence of a benign enzyme-elevating virus. In *Methods in Cancer Research*, (Ed. Busch). New York: Academic Press. p. 493.

RILEY, V., SPACKMAN, D. M., SANTSTEBAN, G. A. & 11 others (1978) The LDH virus: An interfering biological contaminant. *Science*, **200**, 124.

RILEY, V. & WRÓBLEWSKI, F. (1960) Serial lactic dehydrogenase activity in plasma of mice with growing or regressing tumours. *Science*, **132**, 151.

ROBINSON, B. V. & ROBSON, J. M. (1964) Production of an anti-inflammatory substance at a site of inflammation. *Br. J. Pharmacol.*, **23**, 420.

ROWSON, K. E. K. & MAHY, B. W. J. (1975) Lactic dehydrogenase virus. In *Virol. Monogr.*, **13**.

SAITO, H. & TOMIOKA, H. (1980) Suppressive factor of tumour origin against macrophage phagocytosis of *Staphylococcus aureus*. *Br. J. Cancer*, **41**, 259.

SCOTT, M. T. & MILAS, L. (1977) The distribution and persistence *in vivo* of *Corynebacterium parvum* in relation to its antitumour activity. *Cancer Res.*, **37**, 1673.

SNYDERMAN, R. & CIANCIOLO, G. J. (1979) Further studies of a macrophage chemotaxis inhibitor (MCI) produced by neoplasms: Murine tumours free of lactic dehydrogenase virus produce MCI. *J. Reticuloendothel. Soc.*, **26**, 453.

SNYDERMAN, R. & PIKE, M. C. (1976) An inhibitor of macrophage chemotaxis produced by neoplasms. *Science*, **192**, 370.

SNYDERMAN, R., PIKE, M. C., BLAYLOCK, B. & WEINSTEIN, P. (1976) Effect of neoplasms on inflammation: Depression of macrophage accumulation following tumour implantation. *J. Immunol.*, **116**, 585.

SPECTER, S. & FRIEDMAN, H. (1978) Viruses and the immune response. *Pharmacol. Ther.*, *A*, **2**, 595.

TJIO, J. H. & LEVAN, A. (1954) Chromosome analysis of three hyperdiploid ascites tumours of the mouse. *Acta Univ. Lund.*, **50**, 3.

WARR, G. W. & ŠLJIVIĆ, V. S. (1974) Studies on the organ uptake of $^{51}$Cr-labelled sheep erythrocytes in the evaluation of stimulation of RES phagocytic function in the mouse. *J. Reticuloendothel. Soc.*, **16**, 193.

WILKINSON, P. C., O'NEILL, G. J. & WAPSHAW, K. G. (1973) Role of anaerobic coryneforms in specific and non-specific immunological reactions. II. Production of a chemotactic factor specific for macrophages. *Immunology*, **24**, 997.

WYLLIE, A. H., KERR, J. F. R. & CURRIE, A. R. (1980) Cell death: the significance of apoptosis. *Int. Rev. Cytol.*, **68**, 251.